# Screening and Identification of Novel Potential Biomarkers for Breast Cancer Brain Metastases

**DOI:** 10.3389/fonc.2021.784096

**Published:** 2022-01-13

**Authors:** Lulu Wang, Dan Zeng, Qi Wang, Li Liu, Tao Lu, Yan Gao

**Affiliations:** ^1^ Department of Human Anatomy, School of Basic Medical Sciences, Capital Medical University, Beijing, China; ^2^ Beijing Key Laboratory of Cancer Invasion and Metastasis Research, Beijing, China; ^3^ Cancer Institute of Capital Medical University, Beijing, China; ^4^ Department of Experimental Center for Basic Medical Teaching, School of Basic Medical Sciences, Capital Medical University, Beijing, China

**Keywords:** breast cancer, brain metastases, KRT19, FKBP10, GSK3B, SPANXB1

## Abstract

Brain metastases represent a major cause of mortality among patients with breast cancer, and few effective targeted treatment options are currently available. Development of new biomarkers and therapeutic targets for breast cancer brain metastases (BCBM) is therefore urgently needed. In this study, we compared the gene expression profiles of the brain metastatic cell line MDA-MB-231-BR (231-BR) and its parental MDA-MB-231, and identified a total of 84 genes in the primary screening through a series of bioinformatic analyses, including construction of protein-protein interaction (PPI) networks by STRING database, identification of hub genes by applying of MCODE and Cytohubba algorithms, identification of leading-edge subsets of Gene Set Enrichment Analysis (GSEA), and identification of most up-regulated genes. Eight genes were identified as candidate genes due to their elevated expression in brain metastatic 231-BR cells and prognostic values in patients with BCBM. Then we knocked down the eight individual candidate genes in 231-BR cells and evaluated their impact on cell migration through a wound-healing assay, and four of them (KRT19, FKBP10, GSK3B and SPANXB1) were finally identified as key genes. Furthermore, the expression of individual key genes showed a correlation with the infiltration of major immune cells in the brain tumor microenvironment (TME) as analyzed by Tumor Immune Estimation Resource (TIMER) and Gene Expression Profiling Interactive Analysis (GEPIA), suggesting possible roles of them in regulation of the tumor immune response in TME. Therefore, the present work may provide new potential biomarkers for BCBM. Additionally, using GSEA, Gene Ontology (GO) and Kyoto Encyclopedia of Genes and Genomes (KEGG) Enrichment Analysis, we determined the top enriched cellular functions or pathways in 231-BR cells, which may help better understand the biology governing the development and progression of BCBM.

## Introduction

Brain metastases are the most common malignant brain tumors and are the next frontier for the management of metastatic cancer patients (1, 2). Even small lesions can cause neurological disability, and the median survival of patients with brain metastases is less than 1 year (3–5). Breast cancer is the second most frequent cause of brain metastases (3–5), and recently it has surpassed lung cancer as the leading cause of global cancer incidence (6). Despite major advances over past decades in prolonging breast cancer survival, breast cancer brain metastases (BCBM) remain incurable with current therapies, and the incidence is steadily increasing (6, 7). The cumulative incidence of identified brain metastases among patients with breast cancer (all stages at diagnosis) was not high (about 5.1%); however, it varies by subtype. Patients with HER2-positive (34% to 55%) or triple-negative (22% to 46%) subtypes experience significantly higher brain metastasis occurrence than patients with other subtypes (8–10). Moreover, prognosis after brain metastases is also subtype-dependent, and triple-negative breast cancer (TNBC) patients showed the shortest survival time after brain metastasis than other subtypes, which is only 4.9 months (9, 11).

Currently, treatment options for brain metastases include surgery, whole-brain radiotherapy, stereotactic radiosurgery, and systemic drug therapy, such as chemotherapy, targeted therapies, and immunotherapy (12–15). While systemic chemotherapy has limited efficacy, targeted therapies have recently shown promise for BCBM management (16–21). HER2-targeted therapies have been shown to increase the time to development of brain metastases and improved survival following brain metastases (16, 18–21), and patients with estrogen receptor-positive BCBM can be treated with endocrine agents, cyclin-dependent kinases 4/6 (CDK4/6) inhibitors, and the mechanistic target of rapamycin kinase (mTOR) inhibitors (17). Unfortunately, there is no effective targeted therapy for TNBC brain metastases (5, 9, 22). It is desirable to identify potential therapeutic targets or molecular risk factors or early biomarkers for this lethal disease.

Several brain metastasis-related genes and signaling pathways have been identified, such as COX2, PTGS2, HBEGF, ST6GALNAC5, CXCR4, GABA, heparinase, etc. (23–26). However, the molecular basis for BCBM remains largely unknown. The human MDA-MB-231-BR “brain-seeking” breast cancer cell line (hereafter referred to as 231-BR cells) was initially established from the TNBC cell line MDA-MB-231 (27, 28). It metastasizes with 100% frequency to the brain and has been used as an established preclinical model of brain metastatic breast cancer (29–31). In this study, we compared the gene expression profiles of the two cell lines with RNA-sequencing, and carried out a series of bioinformatic analyses and wet-lab experiments to identify the potential genes that may serve as prognostic biomarkers or therapeutic targets for BCBM.

There are many approaches to identify key genes. To be as comprehensive as possible, here we integrate different bioinformatic approaches and obtained a total of 84 differentially expressed genes (DEGs) from the primary screening. Among them, we selected 8 genes that have not been reported to be associated with BCBM in previous studies, and validated their expression levels using quantitative RT-PCR. Following this, we knocked down the 8 genes in 231-BR cells to evaluate their effects on cell migration, and finally 4 genes were identified as key genes for further exploration in our study. The key genes identified here are screened from TNBC cells and showed an impact on migration of the brain metastatic cell line 231-BR. In addition, the overexpression of the above genes was associated with worse distant metastasis–free survival (DMFS) of TNBC patients on data from Gene Expression Omnibus (GEO) database. All these pieces of evidence point to our key genes as potential therapeutic targets in TNBC brain metastases.

To establish a better understanding of the function of the selected genes, we further evaluated their RNA expression on data from The Cancer Genome Atlas (TCGA), evaluated their protein expression on data from the Clinical Proteomic Tumor Analysis Consortium (CPTAC) and HPA database, investigated the relationship between their expression and immune cell infiltration through Tumor Immune Estimation Resource (TIMER) and Gene Expression Profiling Interactive Analysis (GEPIA). It is hoped that these multiple investigative approaches could help decipher the underlying mechanisms of the specific functions of these genes in BCBM. Along with the above, we determined the top differentially regulated pathways using Gene Set Enrichment Analysis (GSEA), Gene Ontology (GO) and Kyoto Encyclopedia of Genes and Genomes (KEGG) Enrichment Analysis to better understand the biology governing the development and progression of BCBM (for a list of abbreviations used in the main text, see **Supplementary Table 1**).

## Materials and Methods

### Cell Culture

The 231-BR cell line was a generous gift from Dr. Patricia Steeg (National Cancer Institute, Bethesda, MD, USA) (27). It was maintained in Dulbecco’s Modified Eagle Medium (DMEM), supplemented with 200 μg/mL G418 (Sigma-Aldrich), 10% Fetal Bovine Serum (FBS), and 1% penicillin-streptomycin. MDA-MB-231 was purchased from ATCC and maintained in DMEM supplemented with 10% FBS and 1% penicillin-streptomycin.

### Transwell Cell Migration and Invasion Assay

Cell migration and invasion assay were performed using 24-well transwell chambers and BioCoat Matrigel invasion chambers (BD Biosciences), respectively. For this, 2×10^4^ cells were suspended in 0.1 ml medium without FBS and added to the upper compartment of the Transwell chamber. Next, 0.6 ml medium with 1% FBS was added to the lower compartment as a chemoattractant. After incubation at 37°C and 5% CO_2_ for 12 h, the cells on the upper surface of the membrane were carefully removed using a cotton bud; and cells on the lower surface were fixed with 70% ethanol for 10 minutes and stained with 0.2% crystal violet. Six fields were randomly selected of each insert at a magnification of 200× with a light microscope (Olympus, Japan). Student’s t-test was used to test for significance. P values of < 0.05 were defined as significant.

### RNA Extraction

Total RNA from 231-BR and MDA-MB-231 cells was extracted using TRIzol reagent (Qiagen, CA, USA) according to the manufacturer’s instructions. Each group was prepared with three parallel replicates. RNA quantity and purity were assessed by a NanoDrop spectrophotometer (Thermo Fisher Scientific, Wilmington, DE, USA).

### RNA Sequencing

RNA sequencing library preparation and sequencing were conducted in BGI Tech (Shenzhen, China) *via* BGISEQ-500 sequencer. The sequencing data was filtered with SOAPnuke (v1.5.2) (https://github.com/BGI-flexlab/SOAPnuke) (32) by removing reads containing sequencing adapter, removing reads whose low-quality base ratio (base quality less than or equal to 5) is more than 20%, and removing reads whose unknown base (‘N’ base) ratio is more than 5%. After this, clean reads were obtained and stored in FASTQ format. The clean reads were mapped to the reference genome using HISAT2 (v2.0.4). Bowtie2 (v2.2.5) was applied to align the clean reads to the gene set, a database built by BGI Tech, with known and novel coding transcripts included (33). The expression level of gene was measured in the normalized read count (given by Fragments Per Kilobase of transcript per Million mapped reads, FPKM). The gene expression heatmap was drawn by pheatmap (v1.0.8). Differential expression analysis was performed using the DESeq2 (v1.4.5) (34). False discovery rate (FDR) adjusted P values (Q value) of < 0.05 were defined as significant. The RNA sequencing data have been uploaded to the GEO with accession number: GSE183862.

### GO and KEGG Enrichment Analysis

In order to gain a better insight to the change of phenotype, GO (including biological processes (BP), molecular functions (MF), and cellular components (CC)) and KEGG enrichment analysis of DEGs were performed using DR.TOM system of BGI Tech as previously described (35). The significant terms and pathways were obtained with a criterion of Bonferroni adjusted P value (Q value) < 0.05. Only the top twenty terms for each category were shown.

### PPI Network Construction and Module Analysis

The PPI (Protein-Protein Interaction) Network of DEGs was constructed using STRING database (version 11.0), and the minimum required interaction score was 0.4 (36). Cytoscape (version 3.7.2) was employed to visualize the molecular interaction networks (37). The MCODE algorithm was used to determine the most significant clusters of highly interacting nodes within the PPI network. The criteria for cluster finding were as follows: MCODE scores > 5, degree cutoff = 2, node score cutoff = 0.2, k-score = 2, and max. depth=100 (38). The CytoHubba algorithm was used to determine the top 30 nodes ranked by Degree in the PPI network (39).

### GSEA

GSEA on RNA-seq expression data was performed using GSEA official software package (https://www.gsea-msigdb.org/gsea/index.jsp). Analyses were performed to identify gene sets that were enriched in 231-BR cells relative to 231 cells. GSEA statistical significance was assessed using GSEA software that calculated FDR. Gene sets were considered significantly enriched if their FDR adjusted P values were less than 0.25, as defined by the publishers of the GSEA tool (40, 41).

### Quantitative RT-PCR

After total RNA was extracted, quantitative RT-PCR was performed using SuperReal PreMix Plus (SYBR Green) (TIGANGEN, Beijing, China) in a final volume of 20 μl containing 10 μM each of the forward and reverse primers as described by the manufacture. Relative levels of transcript expression were measured using CFX96 Real-time System, C1000 Thermal Cycler (BioRad). The relative expression was calculated using the 2−ddct method with GAPDH as endogenous controls. The following primers were used: see **Supplementary Table 2**. Student’s t-test was used to test for significance. P values of < 0.05 were defined as significant.

### RNA Interference Assay

To knock down each candidate gene in 231-BR cells, the lentiviral vector (U6-MCS-Ubiquitin-Cherry-IRES-puromycin) containing the short-hairpin RNA (shRNA) specifically targeting each gene was constructed (GeneChem, China). For lentivirus infection, three individual shRNA oligos targeting each gene were pooled together: see **Supplementary Table 3**, and the HitransG (Genechem) was used according to the manufacturer’s instructions. Student’s t-test was used to test for significance. P values of < 0.05 were defined as significant.

### Wound Healing Assay

The monolayer culture growth rate was determined using a Cellomics Arrayscan (Genechem). Briefly, after infected by lentivirus, cells of the same density were seeded into flat-bottom 96-well plates and grown under normal conditions. Images of the same area were captured at 0, 16 and 24 hours after the scratch using a Cellomics Arrayscan according to the manufacturer’s instructions (GeneChem). The migration area was measured on the images using ImageJ. The wound healing rate was calculated as the area of original wound minus the area of wound during healing divided by the area of original wound. Student’s t-test was used to test for significance. P values of < 0.05 were defined as significant.

### Metastasis-Free Survival Analysis

The metastasis-free survival in breast cancer patients was analyzed on datasets obtained from GEO database through PROGgene Version 2, a comprehensive survival analysis tool (42). Patients were divided into two groups based on the cutoff of median or 25^th^ percentile. Survival analysis was performed using cox proportional hazards analysis. The two groups were compared by a Kaplan-Meier survival plot, and the HR and log rank P value were calculated. The P value was calculated by log rank test. P values of < 0.05 were defined as significant.

### UALCAN Analysis

RNA-Seq-derived gene expression levels from TCGA and protein expression levels from CPTAC were acquired and analyzed by UALCAN portal (http://ualcan.path.uab.edu). The expression levels of the genes were analyzed based on sample types and tumor stages (43). Student’s t-test was used to test for significance. P values of < 0.05 were defined as significant.

### Receiver Operating Characteristic Analysis

ROC analyses were performed in TCGA data using the function “roc” in the R package pROC.

### TIMER Analysis

Correlations between the key genes (KRT19, FKBP10 and GSK3B) expression level and infiltration of immune cells and tumor purity based on TCGA database were calculated and plotted using TIMER2.0 (44, 45). The “Immune-Gene” module were selected, and the TIMER, EPIC, quanTIseq, xCell, MCP-counter, CIBERSORT and CIBERSORT-ABS algorithms were applied for immune infiltration estimations. The correlation coefficient was determined by the Spearman method. P values for the Kaplan-Meier analyses are based on log rank tests.

### GEPIA Analysis

The correlations between gene expression and different immune cell biomarkers were analyzed though GEPIA (http://gepia.cancer-pku.cn/), which is a newly developed interactive web server for analyzing the RNA sequencing expression data of tumors and normal samples from the TCGA and the GTEx projects, using a standard processing pipeline (46). The correlation coefficient was determined by the Spearman method.

### Statistical Analysis

Statistical analyses are described in detail in the respective *Materials and Methods* sections above and in the figure legends. The statistical test is also indicated whenever a P value is reported in the text. Unless specified otherwise, statistical comparisons were performed using GraphPad Prism 7 software.

## Results

### Screening and Identifying of DEGs Based on RNA Sequencing

**Figure 1:**
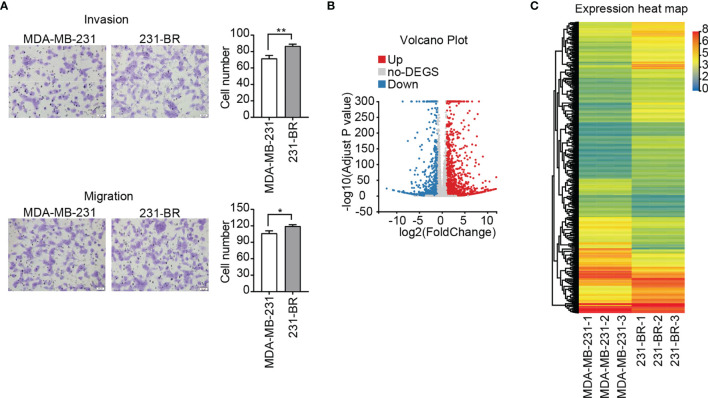
Invasion, migration and gene expression of MDA-MB-231 and 231-BR cells. **(A)** Representative images of invasion and migration assays are shown on the left and quantified data on the right. Bar, 50μm. Student’s t-test was used for statistical analysis: *P < 0.05, **P < 0.01. **(B)** Volcano plot of DEGs. **(C)** The heatmap represents the expression values (FPKM) of DEGs.

To study the characteristics of the brain metastatic variant 231-BR cells, transwell cell migration and invasion assay were performed. As a “brain-seeking” breast cancer cell line, 231-BR cells exhibited an increased invasion and migration capacities as compared with its parental MDA-MB-231 cells, especially the former (**Figure 1A**). In the search for novel genes related to the pathogenesis of breast cancer brain metastasis, DEGs between 231-BR and MDA-MB-231 cells were screened and identified by RNA sequencing. Expression was measured using FPKM. The mRNAs were identified as DEGs if they met the following criteria: the FPKM values≥ 1, FDR adjusted P value (Q value) < 0.05 and |Log2 (fold-change)| > 1. On the basis of this definition, 545 upregulated genes and 315 downregulated genes were identified and shown in volcano plot and heatmap (**Figures 1B, C**).

### KEGG and GO Enrichment Analysis of DEGs

**Figure 2:**
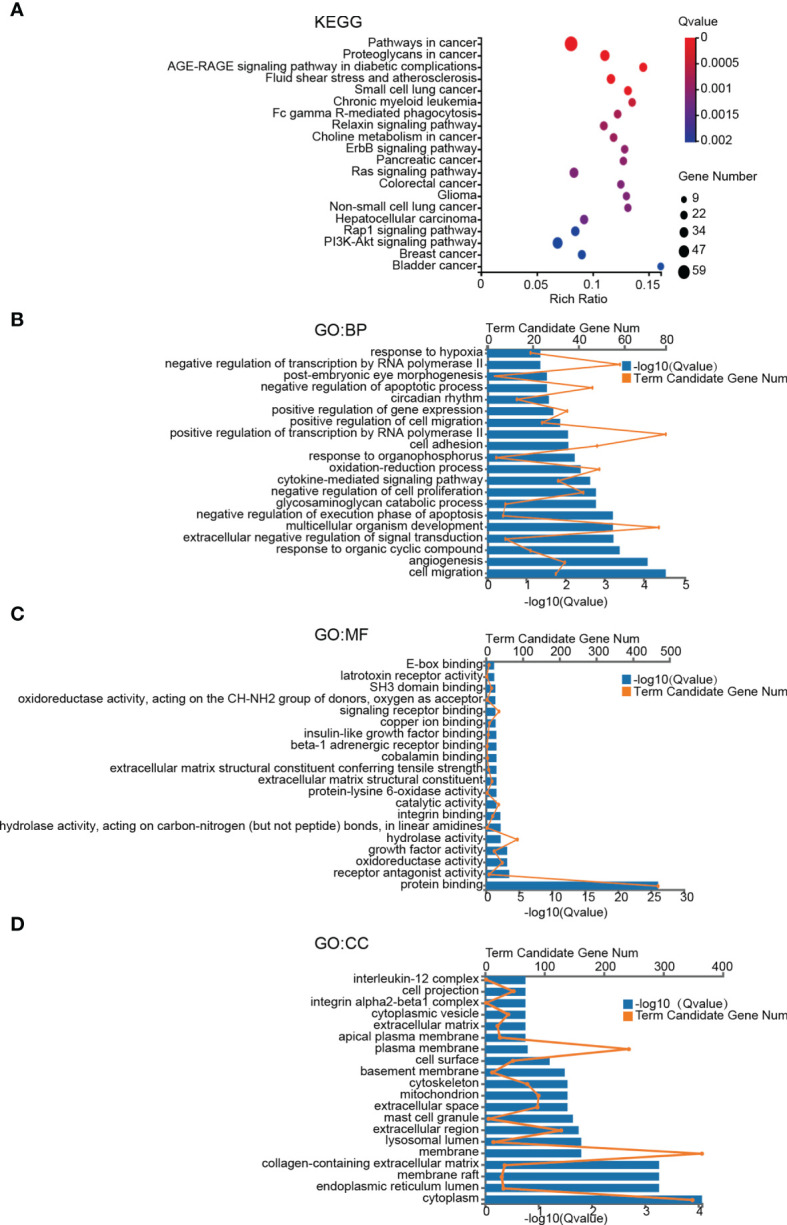
KEGG pathway and GO term enrichment analyses of DEGs. **(A)** KEGG pathway analysis of DEGs. **(B–D)** Enriched GO-terms for BP, MF, and CC. The top twenty terms for each category are shown. The significant pathways and terms were obtained with a criterion of Bonferroni adjusted P value (Q value) < 0.05.

To gain a better insight of the potential mechanisms underlying brain metastases of breast cancer cells, GO and KEGG enrichment analysis of DEGs was performed. The pathway with a criterion of Bonferroni adjusted P value (Q value) < 0.05 was identified as significant. The most significant KEGG pathways are shown in **Figure 2A**. Among the twenty involved pathways, eleven of them were related to Cancers (KEGG Pathway Term Level 2), including Pathways in cancer, Proteoglycans in cancer, Small cell lung cancer, Chronic myeloid leukemia, Choline metabolism in cancer, Pancreatic cancer, Colorectal cancer, Glioma, Non-small cell lung cancer, Hepatocellular carcinoma, Breast cancer and Bladder cancer; four were related to Signal transduction (KEGG Pathway Term Level 2), including ErbB signaling pathway, Ras signaling pathway, Rap1 signaling pathway and PI3K-Akt signaling pathway; two were related to Endocrine system, including AGE-RAGE signaling pathway in diabetic complications and Relaxin signaling pathway; Fluid shear stress and atherosclerosis that was related to Cardiovascular diseases (KEGG Pathway Term Level 2) and Fc gamma R-mediated phagocytosis that was related to Organismal Systems (KEGG Pathway Term Level 2).

GO functional annotation analysis including biological process (BP), molecular function (MF), cellular component (CC) was used to further investigate functional differences of the DEGs. The top 20 most enriched terms of BP, MF and CC were presented in **Figures 2B–D**. Cell migration, angiogenesis and response to organic cyclic compound in BP category, protein binding, receptor antagonist activity and oxidoreductase activity in MF category, and plasma membrane, membrane and integral component of plasma membrane in CC category were the top 3 most significant terms in the 3 categories of GO, respectively (**Figures 2B–D**).

### PPI Network Construction and Module Analysis

**Figure 3:**
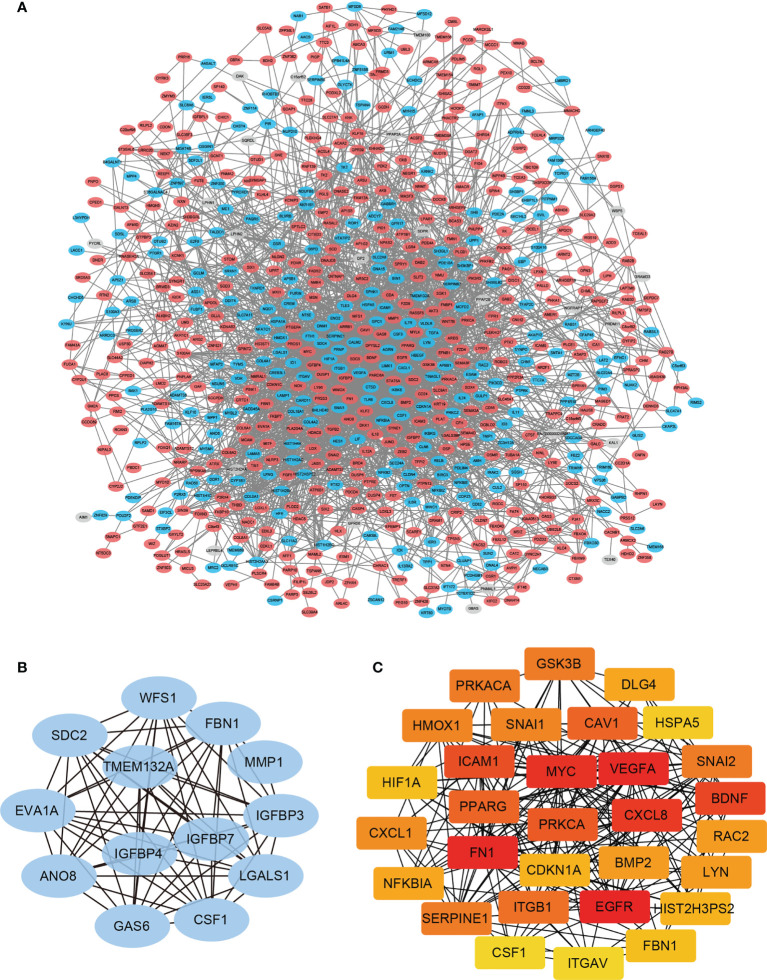
PPI network construction and module analysis. **(A)** The PPI Network of DEGs was constructed using STRING database, and visualized with Cytoscape. **(B)** The most significant cluster of highly interacting nodes within the PPI network as determined by MCODE algorithm. **(C)** The top 30 nodes ranked by degree in the PPI network determined by CytoHubba algorithm.

Hub genes defined as highly interconnected genes in the network have been considered functionally significant. To find the hub genes and clarify the interactions between the DEGs, the PPI network of the 860 DEGs was constructed using STRING database (**Figure 3A**). Two plug-ins of Cytoscape were employed to identify the hub genes: (1) The core network modules of the PPI network were identified by plug-in MCODE of Cytoscape, and the top one significant module with 13 nodes and 68 edges were extracted (Score=11.333). These 13 hub genes were identified and assigned to MCODE Group (**Figure 3B** and **Table 1**). (2) The top 30 nodes ranked by Degree in the PPI network were calculated by the plug-in CytoHubba, and these 30 genes were selected and assigned to CytoHubba Group (**Figure 3C** and **Table 1**).

**Table 1:** Candidate genes from the primary screen.

Group names	Number of genes	Gene symbols
MCODE group	13	CSF1 FBN1 MMP1 IGFBP3 IGFBP4 ANO8 GAS6 LGALS1 WFS1 TMEM132A SDC2 EVA1A IGFBP7
CYTOHUBBA group	30	CSF1 FBN1 GSK3B BDNF LYN NFKBIA HSPA5 CXCL1 PPARG SERPINE1 SNAI1 HIF1A PRKCA PRKACA RAC2 HMOX1 VEGFA DLG4 CXCL8 HIST2H3PS2 EGFR ICAM1 MYC FN1 CAV1 CDKN1A ITGB1 BMP2 SNAI2 ITGAV
GSEA group	36	GSK3B SEMA3A EPHA5 NTN4 NGEF SEMA6D CFL2 EPHA7 EFNB1 NFATC4 SEMA3E DCC CXCR4 EPHA4 FES SLIT2 ROBO2 EFNA3 EPHA6 SEMA4G EPHA3 ROBO3 PLXNC1 EPHB1 SEMA3B SRGAP3 SEMA3G GNAI1 ABLIM1 EPHA1 SEMA3F SEMA4D FYN DPYSL2 UNC5C SRGAP1
TOP DEGs group	10	MMP1 SEMA3A KRT19 SOCS2 SPANXB1 FST KCNAB2 FKBP10 MYO1D ESM1

### Pathway Enrichment Assessed by GSEA

**Figure 4:**
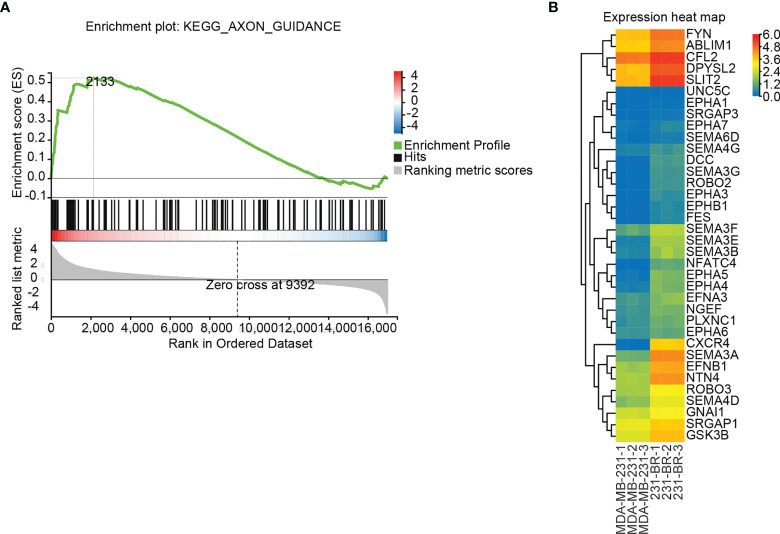
Differentially regulated pathways determined using GSEA. **(A)** GSEA identified the “Axon guidance” signaling pathway as significant (FDR adjusted P value < 0.25). **(B)** Expression values (FPKM) of the 36 leading edge subsets of “Axon guidance” pathway are shown in a heatmap.

GSEA is a computational method to determine the statistical significance of *a priori* defined set of genes and the existence of concordant differences between two biological states (40, 41). Upon performing the GSEA analysis, Axon guidance was the only significant signaling pathway identified by the default setting in the GSEA tool, with FDR P value = 0.114, Nominal P value < 0.0001, Normalized Enrichment Score (NES) = 1.715, ES = 0.523, Leading edge: tags=29%, list=13%, signal=32%, FWER P value: 0.129 (**Figure 4A**). The elevated expression of the 36 leading edge subsets in 231-BR groups was shown (**Figure 4B**), and these genes were assigned to the GSEA Group (**Table 1**).

### Identification of Candidate Genes

From the primary screening, genes that may be related to brain metastatic potential were determined through the following approaches and assigned to four groups accordingly: (1) MCODE Group: the 13 hub genes identified by MCODE (**Figure 3B**, **Table 1** and **Supplementary Table 4**); (2) Cytohubba Group: the 30 hub genes identified by Cytohubba (**Figure 3C**, **Table 1** and **Supplementary Table 5**); (3) GSEA group: the 36 leading edge subsets of GSEA (**Figure 4B**, **Table 1** and **Supplementary Tables 6**, **7**); (4) TOP DEGs group: the 10 most up-regulated DEGs in 231-BR group determined by Log2 (fold-change) (**Table 1** and **Supplementary Table 8**). Accordingly, a total of 84 unique genes were identified (**Table 1**).

**Figure 5:**
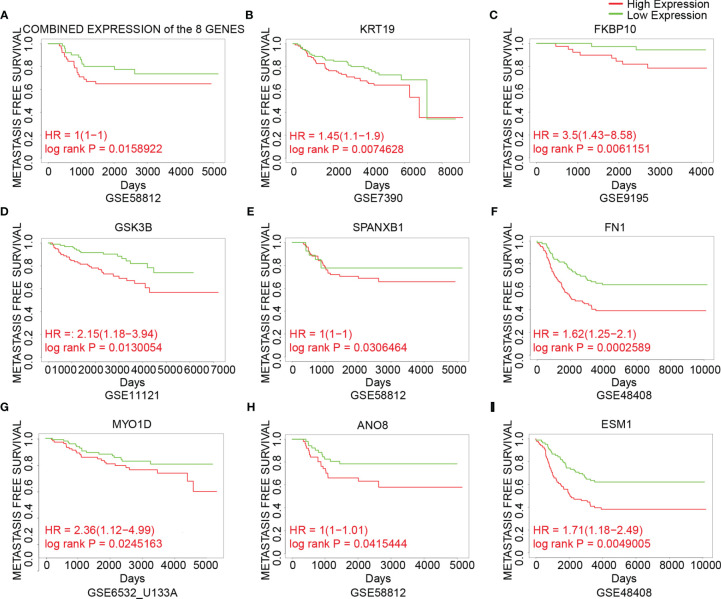
The association between expression of the candidate genes and metastasis-free survival in breast cancer. **(A)** The association between the combined expression of the 8 candidate genes and metastasis-free survival in breast cancer cohorts. **(B–I)** The association between expression of individual genes and metastasis-free survival in breast cancer cohorts. Samples were obtained from the GEO database. The P value was calculated by log rank test.

To analyze the prognostic value of the genes, the correlation between gene expression and metastasis-free survival, which was defined as time from diagnosis to distant metastasis as first event, was assessed using datasets from GEO (**Figure 5**). Patients were divided into two groups based on the cutoff of median or 25th percentile. High expression of combined expression of the 8 genes, KRT19, FKBP10, GSK3B, SPANXB1, FN1, MYO1D, ANO8 and ESM1 in breast cancer patients was associated with worse metastasis-free survival in breast cancer patients (combined expression: log rank P = 0.0159, HR = 1; KRT19: log rank P = 0.0075, HR = 1.45; FKBP10: log rank P = 0.0061, HR = 3.5; GSK3B: log rank P = 0.0130, HR = 2.15; SPANXB1: log rank P = 0.0306, HR = 1; FN1: log rank P = 0.0003, HR = 1.62; MYO1D: log rank P = 0.0245, HR = 2.36; ANO8: log rank P = 0.0415, HR = 1; ESM1: log rank P = 0.0049, HR = 1.71) (**Figure 5**). Meanwhile, their roles in BCBM have not been thoroughly investigated in previous studies. Therefore, the above 8 genes were identified as our candidate genes (**Figure 5**).

**Figure 6:**
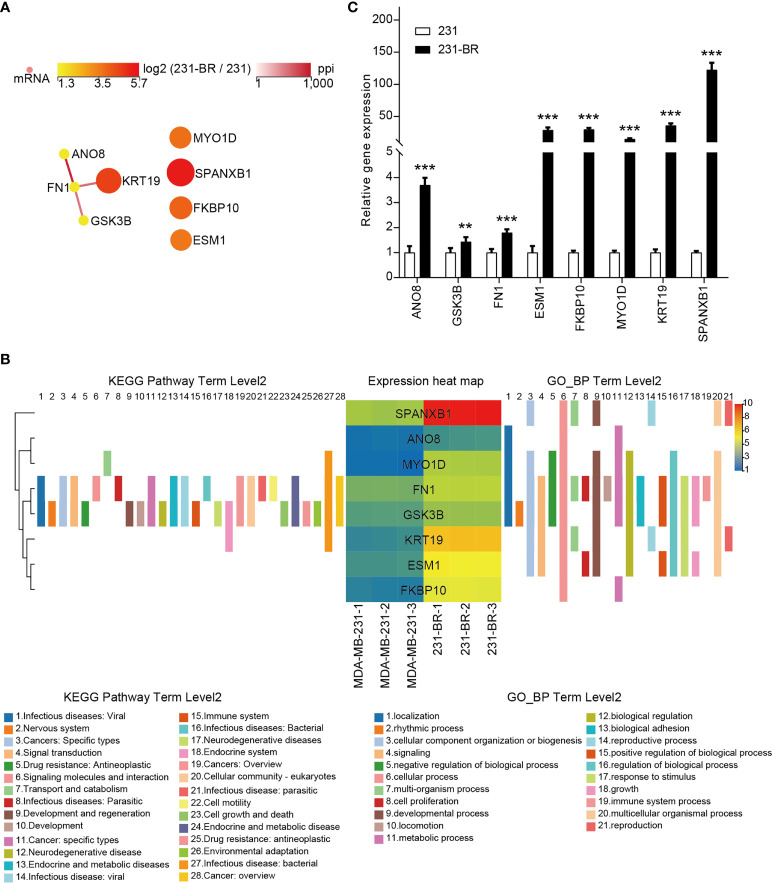
The 8 candidate genes selected from the screening. **(A)** The PPI network of the 8 candidate genes. **(B)** The KEGG pathway annotations and GP_BP terms of the 8 candidate genes. **(C)** The mRNA levels of all candidates were validated by quantitative RT-PCR. Student’s t-test was used for statistical analysis: **P < 0.01, ***P < 0.001 compared with 231 group.

The PPI network of the candidate genes was built with STRING and showed in **Figure 6A**. They were annotated using KEGG pathway annotations and GO terms (**Figure 6B** and **Supplementary Figure 1**). The expression levels of the candidate genes were verified using quantitative RT-PCR (**Figure 6C**). These genes all showed a higher expression in 231-BR cells as compared with MDA-MB-231 cells.

### The Prognostic Values of the Candidate Genes in BCBM and Effects of Them on 231-BR Cell Migration

**Figure 7:**
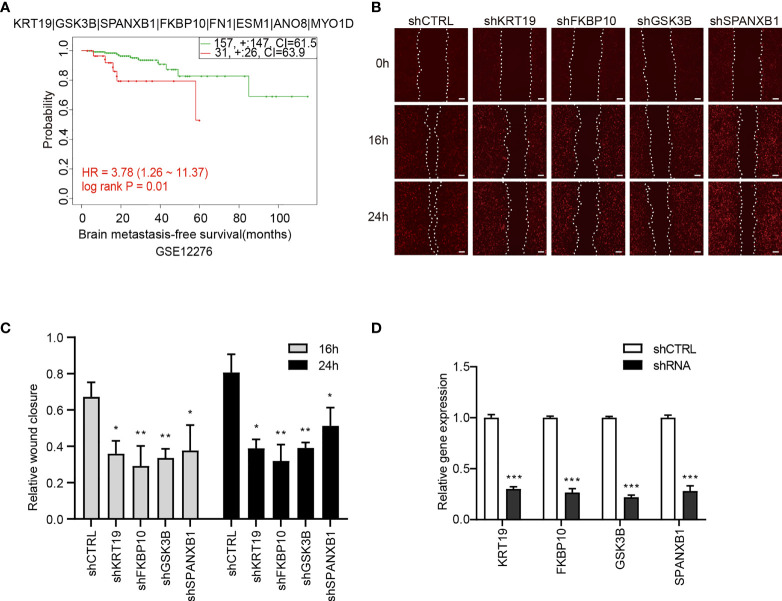
The association of the candidate gene set with brain metastasis-free survival of breast cancer patients and effects of the genes on migration of 231-BR cells. **(A)** The association between the candidate gene set and brain metastasis-free survival in breast cancer patients. Samples were obtained from the GEO database. The P value was calculated by log rank test. **(B)** Knockdown of KRT19, FKBP10, GSK3B and SPANXB1 inhibited the migration of 231-BR cells as determined by wound healing assay, and the representative images are shown. Bar, 200μm. **(C)** The quantified data of wound healing assay are shown. Student’s t-test was used for statistical analysis: *P < 0.05, **P < 0.01, ***P < 0.001 compared with corresponding control (shCTRL) at the same time point. **(D)** Knockdown efficiency of shRNAs was verified by quantitative RT-PCR. Student’s t-test was used for statistical analysis: ***P < 0.001 compared with shCTRL.

To explore the prognostic values of the candidate genes in BCBM, we analyzed the relationship of the gene expression with brain-metastasis survival in breast cancer patients using data from a public dataset GSE12276, which contain the brain relapse information of a total of 204 patients. We assessed the prognostic value using Cox proportional hazards analysis, with risk group as covariate and brain metastasis-free survival as endpoint. The candidate gene set showed a significantly correlation with brain metastasis-free survival of breast cancer patients [log rank P = 0.011, hazard ratio (HR) = 3.781, CI = 1.257 − 11.368], indicating a prognostic value of the gene set in predicting brain metastasis (**Figure 7A**).

Next, Each gene was analyzed individually for its effect on 231-BR cell migration. The 8 candidate genes were individually knocked down in 231-BR cells, and cell migration was evaluated using the wound healing assay (**Figures 7B, C**). Knockdown efficiency was verified by quantitative RT-PCR (**Figure 7D**). As shown, among the above-mentioned 8 genes, knockdown of four genes: KRT19, FKBP10, GSK3B, and SPANXB1 significantly inhibited the migration of 231-BR cells. Arising from this, these four genes were determined as the final key genes in our study.

### Expression of the Key Genes in Breast Cancer Patients

**Figure 8:**
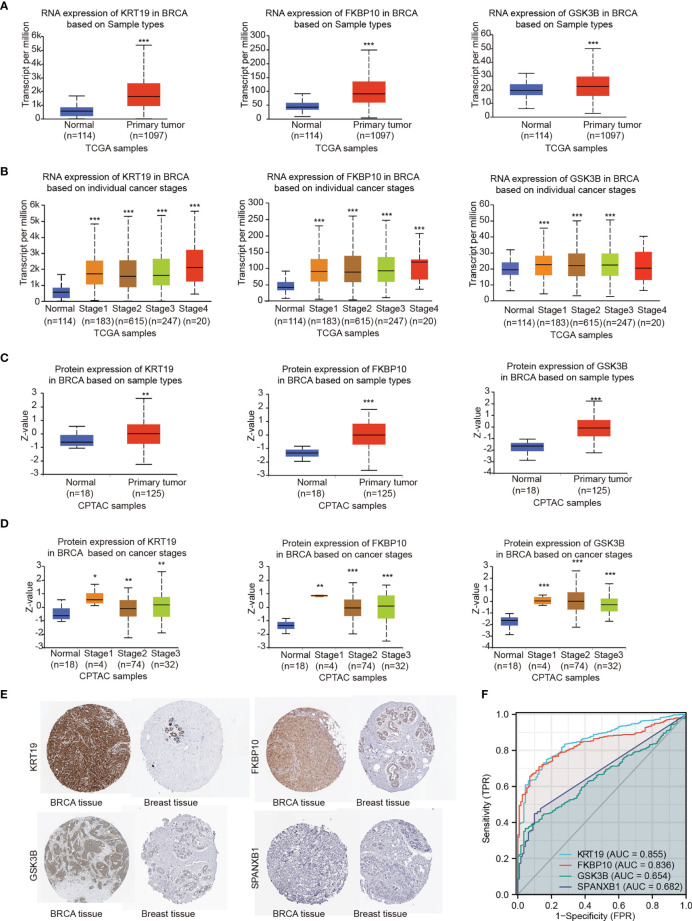
Gene expression levels were explored by TCGA, CPTAC and HPA databases. **(A)** RNA expression levels of KRT19, FKBP10 and GSK3B were explored by TCGA based on sample types. **(B)** RNA expression levels of KRT19, FKBP10 and GSK3B were explored by TCGA based on cancer stages. **(C)** Protein expression levels of KRT19, FKBP10 and GSK3B were explored by CPTAC based on sample types. **(D)** Protein expression levels of KRT19, FKBP10 and GSK3B were explored by CPTAC based on cancer stages. **(E)** Protein expression levels of KRT19, FKBP10 GSK3B and SPANXB1 as detected by immunohistochemistry staining from the HPA database. **(F)** ROC curves of the key genes using data from TCGA. Expression values were compared using Student’s t-test: *P < 0.05, **P < 0.01, ***P < 0.001 compared with Normal.

In the results section above, the candidate gene set showed a significantly correlation with brain metastasis-free survival of breast cancer patients, and each individual candidate genes appear to have an impact on metastasis-free survival of breast cancer patients. In addition, each individual key genes showed an effect on the migration of 231-BR cells (**Figure 7**). These data indicated these genes may serve as potential biomarkers in BCBM. To better understand the functions of the genes in breast cancer, we next evaluated the expression of the key genes in breast cancer patients using Samples from the public databases, including TCGA, CPTAC, and HPA Databases (**Figures 8A–E**).

The RNA and protein expression of the key genes in breast cancer patients based on sample types and individual cancer stages was evaluated using TCGA and CPTAC databases, respectively (**Figures 8A–D**). Elevated expression of KRT19, FKBP10 and GSK3B at both transcriptional and translational levels in BRCA were observed as compared with normal breast tissue, while the transcript per million (TPM) values of SPANXB1 were extremely low (TPM < 1) and not shown. Moreover, HPA database was applied to validate the expression of the key genes at protein level. The similar result was obtained, that is, KRT19, FKBP10 and GSK3B protein all showed elevated expression in BRCA tissue compared with normal breast tissue, whereas SPANXB1 was not detected (**Figure 8E**). The expression levels of the four key genes were also evaluated in human cancer cell lines, particularly in breast cancer cell lines, and overexpression of KRT19, FKBP10 and GSK3B in TCGA breast cancer patients and breast cancer cell lines were observed (**Supplementary Figure 2**). In addition, relationships between KRT19, FKBP10 and GSK3B expression and clinicopathological features from TCGA breast cancer cohort (n = 1083) were also explored (**Supplementary Table 9**). It should be noted that although the expression level of SPANXB1 was very low in breast cancer patients as well as in most of the human cancer cell lines (**Supplementary Figure 2**), it showed a very high expression in the brain metastatic 231-BR cells as compared with its parental MDA-MD-231. In light of its pro-migratory effect on 231-BR cells as well as its prognostic value in metastasis-free survival of breast cancer patients (**Figures 5E**, **7**), SPANXB1 was therefore also considered as a key gene in the present study.

To assess the predictive performance of the key genes in breast cancer, we performed ROC analysis and used the area under the ROC curve (AUC) as an assessment of the prediction accuracy. A total of 1,083 breast tumor samples and 111 normal breast samples were obtained from TCGA. As shown in **Figure 8F**, KRT19 (AUC = 0.855, CI = 0.825 - 0.885) and FKBP10 (AUC = 0.836, CI = 0.808 - 0.864) had a certain accuracy in predicting cancer and normal, and the predictive abilities of GSK3B (AUC = 0.654, CI = 0.613 - 0.696) and SPANXB1 (AUC = 0.682, CI = 0.650 - 0.714) were less accurate.

### Correlation Between Gene Expression and Infiltration of Immune Cells in Breast Cancer

The tumor microenvironment (TME) landscape in brain metastases was analyzed recently, which revealed that breast brain metastases showed the highest neutrophil infiltration of myeloid cells compared with non-tumor, glioma, melanoma brain metastases and lung cancer brain metastases (47). Meanwhile, CD4+ and CD8+ T cells are the major immune cells of lymphocytes in breast brain metastases (47).

**Figure 9:**
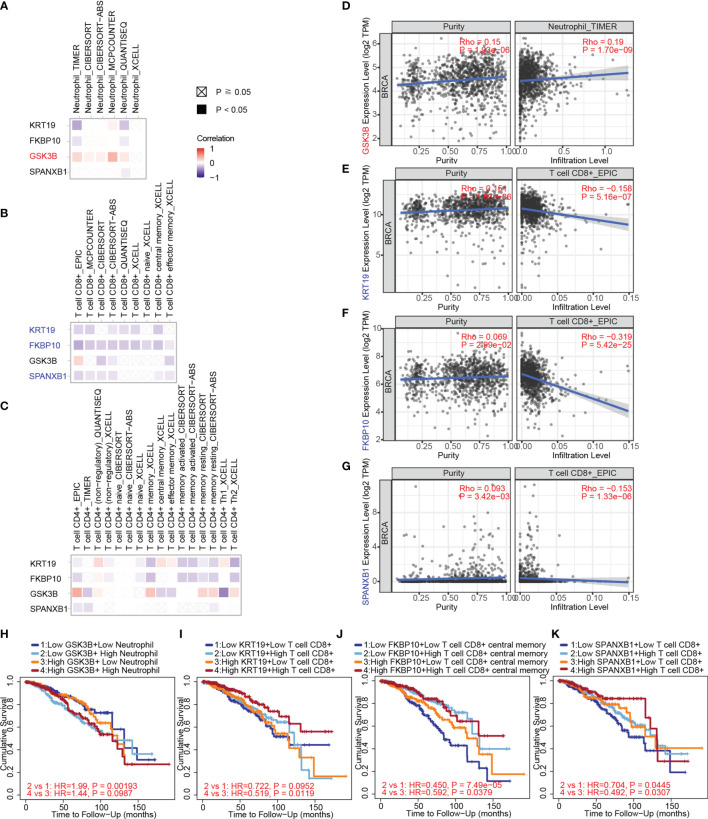
The correlation between key gene expression and immune cell infiltration in breast cancer samples from TCGA through TIMER. **(A)** The correlations between key gene expression and immune infiltration levels of neutrophils are shown in a heatmap. **(B)** The correlations between gene expression and immune infiltration levels of CD8+ T cells. **(C)** The correlations between gene expression and immune infiltration levels of CD4+ T cells. **(D)** The correlations of GSK3B with tumor purity (left) and infiltration level (right) of neutrophils in breast cancer estimated by TIMER. **(E)** The correlations of KRT19 expression with tumor purity and infiltration level of CD8+ T cells in breast cancer estimated by EPIC algorithm. **(F)** The correlations of FKBP10 expression with tumor purity and infiltration level of CD8+ T cells in breast cancer estimated by EPIC algorithm. **(G)** The correlations of SPANXB1 expression with tumor purity and infiltration level of CD8+ T cells in breast cancer estimated by EPIC algorithm. **(H)** The associations of the neutrophil and GSK3B expression levels (high versus low) with patient survival on Kaplan–Meier curves. **(I)** The associations of the CD8+ T cells and KRT19 expression levels (high versus low) with patient survival on Kaplan–Meier curves. **(J)** The associations of the CD8+ T cells and FKBP10 expression levels (high versus low) with patient survival on Kaplan–Meier curves. **(K)** The associations of the CD8+ T cells and SPANXB1 expression levels (high versus low) with patient survival on Kaplan–Meier curves. The correlation coefficient was determined by the Spearman method in **(A–G)**. P values for the Kaplan-Meier analyses are based on log rank tests in **(H–K)**.

Herein, we used TIMER, EPIC, quanTIseq, xCell, MCP-counter, CIBERSORT and CIBERSORT-ABS algorithms to investigate the potential correlations between key gene expression and immune infiltration levels of neutrophils, CD4+ and CD8+ T cells in 1,100 breast cancer samples from TCGA through the TIMER 2.0 web server. The correlation coefficients (Spearman’s Rho values) between the expression of the key genes and the abundance of the immune cell type as well as its subtypes were shown in heatmaps (**Figures 9A–C**). A positive correlation of GSK3B expression with neutrophil infiltration was observed based on most algorithms (**Figure 9A**). The correlations of the above gene with tumor purity and infiltration level of neutrophil in breast cancer estimated by TIMER algorithm were shown in **Figure 9D** (Rho value = 0.19, P value = 1.70e-09).

Negative correlations of KRT19, FKBP10 and SPANXB1 expression with CD8+ T cell infiltration were observed (**Figure 9B**). The correlations of KRT19, FKBP10 and SPANXB1 expression with tumor purity and infiltration level of CD8+ T cell in breast cancer estimated by EPIC algorithm were shown in **Figure 9E** (Rho value = -0.158, P value = 5.16e-07), 9F (Rho value = -0.319, P value = 5.42e-25) and 9G (Rho value = -0.153, P value = 1.33e-06), respectively. Furthermore, how the expression level (high versus low) of the immune cells and the key genes are associated with patient survival on Kaplan–Meier curves were explored (**Figures 9H–K**). Low GSK3B expression with low neutrophil infiltration group has a better survival as compared with low GSK3B expression with high neutrophil infiltration group (**Figure 9H**). Low KRT19, FKBP10 and SPANXB1 expression with low CD8+ T cell infiltration group has a poorer survival as compared with low KRT19, FKBP10 and SPANXB1 expression with high CD8+ T cell infiltration group, whereas high KRT19, FKBP10 and SPANXB1 expression with low CD8+ T cell infiltration group has a poorer survival as compared with high KRT19, FKBP10 and SPANXB1 expression with high CD8+ T cell infiltration group (**Figures 9I–K**).

### Correlation Between Gene Expression and Biomarkers of Different Immune Cell Subsets in Breast Cancer

As microglia (MG), monocyte-derived macrophages (MDMs), neutrophils, and CD8+ and CD4+ T cells have been confirmed to be the major immune cell determinants of the brain TME landscape (47), we investigated the association between the key genes and the above immune cells based on immune biomarkers expression in breast cancer *via* GEPIA. The results indicated a negative correlation between KRT19 expression and expression of CD8+ T cells (biomarkers: CD8A and CD8B), CD4+ T cells (biomarker: CD4), neutrophils (biomarkers: CD66b, CD11b and CCR7), and MDMs (biomarkers: AHR, FCGR2B, CLEC10A, CD1C, CD1B, CD207 and CD209); a negative correlation between FKBP10 expression and expression of CD8+ T cells (biomarkers: CD8A and CD8B); a positive correlation between GSK3B expression and expression of neutrophils (biomarker: CD11b); a negative correlation between SPANXB1 expression and expression of CD8+ T cells (biomarkers: CD8A, CD8B), neutrophils (biomarkers: CD11b and CCR7) and MDMs (biomarkers: AHR, FCGR2B, CLEC10A, CD1C, CD1B, CD207 and CD209) (**Table 2**).

**Table 2:** Correlation between gene expression and biomarkers of different immune cell subsets in breast cancer.

Description	Gene Markers	KRT19		FKBP10		GSK3B		SPANXB1	
		Cor	P value	Cor	P value	Cor	P value	Cor	P value
CD8+ T cell	CD8A	**-0.20**	**2.30e-11**	**-0.19**	**1.40e-10**	-0.05	1.40e-01	**-0.13**	**1.70e-05**
	CD8B	**-0.22**	**1.80e-13**	**-0.20**	**3.10e-11**	**-0.09**	**5.30e-03**	**-0.15**	**8.10e-07**
CD4+ T cell	CD4	**-0.21**	**2.60e-12**	**-0.07**	**2.00e-02**	**0.09**	**2.60e-03**	**-0.09**	**2.30e-03**
Neutrophil	CD66b(CEACAM8)	**-0.08**	**1.20e-02**	-0.05	1.20e-01	0.01	8.20e-01	0.04	1.70e-01
	CD11b(ITGAM)	**-0.11**	**1.80e-04**	0.02	4.40e-01	**0.15**	**1.40e-06**	**-0.06**	**3.60e-02**
	CCR7	**-0.14**	**2.40e-06**	**-0.17**	**1.80e-08**	-0.06	6.60e-02	**-0.13**	**1.40e-05**
MG	P2RY12	**-0.10**	**6.50e-04**	-0.05	1.40e-01	**0.06**	**4.60e-02**	**-0.12**	**6.60e-05**
	TMEM119	-0.05	9.70e-02	**0.10**	**1.40e-03**	-0.03	4.00e-01	**-0.06**	**4.10e-02**
	TAL1	-0.03	3.30e-01	**0.08**	**5.50e-03**	-0.05	1.30e-01	**-0.08**	**1.20e-02**
	SALL1	0.03	3.10e-01	**0.19**	**5.20e-10**	**0.27**	**4.90e-19**	**0.17**	**1.10e-08**
MDMs	AHR	**-0.09**	**1.90e-03**	0.03	3.00e-01	**0.44**	**1.10e-53**	**-0.07**	**2.80e-02**
	FCGR2B	**-0.16**	**1.50e-07**	-0.01	7.50e-01	**0.11**	**4.20e-04**	**-0.07**	**2.10e-02**
	CLEC10A	**-0.16**	**1.40e-07**	**-0.14**	**6.20e-06**	**-0.12**	**1.10e-04**	**-0.18**	**2.7e−09**
	CD1C	**-0.15**	**3.80e-07**	**-0.09**	**2.30e-03**	**-0.11**	**1.70e-04**	**-0.15**	**4.1e−07**
	CD1B	**-0.19**	**1.90e-10**	**-0.17**	**2.50e-08**	**-0.06**	**4.90e-02**	**-0.10**	**1.50e-03**
	CD207	**-0.14**	**1.70e-06**	-0.02	5.50e-01	0.003	9.30e-01	**-0.09**	**3.20e-03**
	CD209	**-0.20**	**6.50e-11**	-0.03	3.40e-01	**0.16**	**1.10e-07**	**-0.14**	**7.5e−06**

Cor, R value of Spearman’s correlation. Bold values indicate P value < 0.05.

To better understand the possible functional states of the key genes in breast cancer, we explored the expression characteristics of the key genes at the single-cell level through CancerSEA, (http://biocc.hrbmu.edu.cn/CancerSEA/), a database that aims to comprehensively decode distinct functional states of cancer cells at single-cell resolution (48). As shown in **Supplementary Figure 3**, KRT19, FKBP10, GSK3B, and SPANXB1 have been investigated at the single-cell level in 9, 10, 16 and 3 types of cancer, respectively (**Supplementary Figures 3A–D**). Correlations between the gene and functional state in different single-cell datasets were filtered by the correlation > 0.3 and P value < 0.05 (Spearman’s rank correlation test with Benjamini & Hochberg FDR correction for multiple comparisons). In breast cancer (GSE77308) (49), KRT19 and SPANXB1 were shown to be correlated with several functional states. KRT19 was positively correlated with metastasis, hypoxia and stemness, and negatively correlated with DNA repair, inflammation, cell cycle, proliferation (**Supplementary Figure 3E**). SPANXB1 was positively correlated with inflammation and proliferation (**Supplementary Figure 3F**).

## Discussion

Attempts to identity new therapeutic targets for BCBM are emerging (23–26). In the present study, we focused on mining RNA-seq data of brain metastatic breast cancer cell lines and multiple clinical cohorts, by utilizing an integrated bioinformatic analyses approach and leveraging a comprehensive collection of databases, we identified potential biomarkers, validated their functions in brain metastatic breast cancer cell migration, and showed their clinical relevance to breast cancer metastasis. Our study not only provided unprecedented insights into BCBM, but also showcased the bioinformatics analytical pipeline that could be applied to other cancers.

Enrichments of Proteoglycans in cancer signaling pathway and Collagen-containing ECM can be observed in our KEGG and GO-CC analyses (**Figures 2A–D**). Therefore, as two main components of the extracellular matrix, which played critical roles in malignant cell behavior and cancer metastasis (50), proteoglycans and collagens may play roles in regulating 231-BR cellular functions. For the latter, collagen fibers can lay tracks for cells to migrate (51, 52), and the remodeled stiff collagens might be exploited as invasion “highways” by cancer cells (51–53). Among the identified key genes in our study, FKBP10 is a molecular chaperone able to pro-collagen maturation in fibroblasts and contributes to high-collagenous ECM (54, 55). For Proteoglycans in cancer pathway, it enables a mesenchymal phenotype with increased cellular motility. Proteoglycans in the ECM can make the extracellular space more compliant for migration, and cell-surface proteoglycans receive signals triggered by interactions with ECM components and modulate cellular behavior such as migration (56–58). There was not much evidence highlighting the relationships between Proteoglycans in cancer pathway and the 4 key genes. However, as one of the 8 candidate genes (**Figures 5**, **6**), which showed a significantly correlation with brain metastasis-free survival as a gene set (**Figure 7A**), FN1 contributes to the “proteoglycans in cancer” pathway (KEGG Pathway Map: 05205). Although FN1 was not identified as key genes in our study because it did not affect 231-BR cell migration in wound healing assay, its possible role in BCBM through Proteoglycans in cancer signaling pathway should not be ignored. This requires investigation in future studies.

GSEA is a computational method to determine whether a predefined set of genes shows statistical difference between two sets of processes or phenotypes (40, 41). Based on our RNA seq data, Axon guidance was the only signaling pathway identified through GSEA (**Figure 4**), indicating a role of it in phenotype determination of 231-BR cells. Axon guidance is a specialized form of cell migratory phenomenon (59) and has been implicated in tumor cell migration (60). Meanwhile, as one of the identified key genes, GSK3B is a member of the above pathway (KEGG Pathway Map: 04360). The emergence of this pathway indicates the regulation of Axon guidance by GSK3B may thereby affect the promigratory phenotype of 231-BR cells. This needs to be demonstrated in future studies.

The brain has been considered previously to be an immune privileged site. Indeed, it had remained uncertain for a long time whether immune cells exist and function in the brain TME (61). Recently, it has been reported that various types of immune cells can be recruited into the brain TME when the blood-brain barrier is compromised by metastatic cancer cells (61). The co-evolution of metastatic cancer cells with the brain microenvironment is critical for metastatic cells’ escaping dormancy and colonizing the brain. The TME landscape in brain metastases was analyzed and MG, MDMs, neutrophils, and CD8+ and CD4+ T cells have been confirmed to be the major immune cell determinants of the brain TME landscape (47, 62). To better predict the functions of the above genes in breast cancer, we explored the correlations between gene expression level and infiltration of immune cells. Meanwhile, correlation between gene expression and biomarkers of different immune cell subsets in breast cancer were explored. As one of the identified key genes, GSK3B positively correlated with neutrophil infiltration (**Figure 9A**). Neutrophils play important and contradictory roles in cancer development. In the TME, they may inhibit tumor progression by generating anti-tumor factors (63). However, more frequently, they are reported as tumor accomplices to promote cancer metastasis (64–67) and seems to be an indicator of poor outcome (68, 69). A common mechanism of how tumors can induce neutrophilia seems to be the production by tumors of cytokines that influence granulopoiesis (70). In breast cancer, neutrophils have been shown to drive metastatic establishment within the lung TME (65), meanwhile, they represented a major immune compartment and showed a high infiltration in the brain TME (47). Therefore, the positive correlation of GSK3B and neutrophil infiltration may suggest a metastasis-promoting effect or a prognostic role or of this gene in BCBM (**Figure 9**).

Three of the identified key genes (FKBP10, KRT19, and SPANXB1) negatively correlated with the infiltration of CD8+ T cells, which is the lymphocytes primarily responsible for immune-mediated tumor cell death (**Figure 9B**). One possible cause of the immunosuppression caused by FKBP10 is that collagen can act as a regulator for tumor associated immune infiltration (71–73). High-fibrillar collagens could act as barrier to immune infiltration, and stop the production of chemokines, that lead to suppression of the anti-tumor immune response in the TME (71–74). Higher collagen deposition resulted in tumor immune suppression characterized by decreased total CD8+ T cells and increased exhausted CD8+ T cell subpopulations due to the leukocyte-specific collagen receptor LAIR1, which suppresses lymphocytic activity and is expressed on CD8+ T cells following integrin beta 2 binding to collagen (71–77). Few studies have explored the effects of the other two (KRT19, and SPANXB1) in immunoregulation. However, interrupting expression of KRT19 in mouse tumors prevented the formation of the CXCL12–KRT19 coating, allowed the accumulation of T cells (78), suggesting a possible role of KRT19 in immunoregulation. Considering low CD8+ T cell infiltration often associated with poor outcome and CD8+ T cell is one of the major immune cell determinants of the brain TME (47, 71, 74), the negative correlation between the identified genes (KRT19, FKBP10 and SPANXB1) and CD8+ T cell infiltration suggests the immunosuppressive and metastasis-promoting effects in BCBM.

To the best of our knowledge, the effects of our key genes on BCBM have not been reported so far. Some previous studies have shown effects of these genes on cell migration or metastasis to other sites in some malignant tumors. KRT19 encodes a protein belonging to the keratin family, which are integrated in the cellular framework and interact with a range of cellular proteins (14, 15). It has been shown to exhibit tumor-promoting effects in breast, hepatocellular carcinoma, oral squamous cell carcinomas and lung cancers (79–81). However, studies in breast cancer cells have shown that modulation of KRT19 expression led to contrasting effects on cell behaviors. It can either suppress cell proliferation, migration and invasion (14, 15, 82), or promote oncogenesis, tumor growth and metastasis (83, 84). MARIA et al. reported that KRT19 was only detected in circulating tumor cells of breast cancer patients, but not in healthy donors. The KRT19-positive detections correlated with the diagnosis and high proliferation rate of breast cancer (85), and the combined positive detection of PTHRP-plus-KRT19 correlated with the presence of distant metastasis, especially with bone metastasis (85). These results also asked whether KRT19 could be a marker in breast cancer bone metastasis, which need further investigation. In addition, KRT19 is involved in Estrogen signaling pathway (KEGG Pathway Map: 04915), which has been shown to stimulate cell migration and contribute to brain metastases of breast cancer (86–89). MDA-MB-231 cells also express estrogen receptors, including wild-type ERα, ERα variants (ERα Δ5 and Δ7) and ERβ variants (ERβ1 and ERβ2) (90–93). Moreover, although not in the top 20, Estrogen signaling pathway was enriched in our KEGG analysis (Term Candidate Gene Num = 20, Q value = 0.02). These findings may help explain why KRT19 was identified as key genes in this study.

FKBP10 is a gene encoding FKBP65, which belongs to the FKBP-type peptidyl-prolyl cis/trans isomerase family. This protein localizes to the endoplasmic reticulum and acts as a molecular chaperone (RefSeq database). FKBP family members are involved in multiple cellular processes, including receptor signaling, protein folding, transcription, chaperone activity and immunosuppression (94). A growing body of evidence has suggested that FKBPs play important roles in cancer (95, 96). FKBP10 has been studied in some cancers and its role is currently controversial (97–101), while few studies have investigated FKBP10 in breast cancer. FKBP10 has been reported to be an intracellular regulatory factor for ECM reconstruction and directly interact with collagen I (54, 55). Combined with our GO-CC results that showed an enrichment of Collagen-containing extracellular matrix (**Figures 2A, D**), the role of FKBP10 in 231-BR cellular behavior may partly be explained.

The protein encoded by GSK3B is a serine-threonine kinase belonging to the glycogen synthase kinase subfamily. It is one of the few signaling mediators that play central roles in a diverse range of signaling pathways, and it has been shown to be involved in energy metabolism, inflammation, apoptotic pathways, ER-stress, and mitochondrial dysfunction (102). Multiple roles have been suggested for GSK3B in different cancers, and even after years of study they remain complex and controversial (103). Due to its ability to phosphorylate and thereby target some pro-oncogenic molecules for ubiquitin-dependent proteosomal degradation, GSK3B has been thought of potential tumor suppressor in some cancers (104–106). However, recent reports have suggested that GSK3B is a positive regulator of cancer progression (107–111). In breast cancer, GSK3B knockdown has been shown to inhibit cell proliferation, and GSK3B overexpression has been shown to correlate with poor prognosis in TNBC patients (112–114).

SPANXB1 is a member of the SPANX family, which consists of five members all located in a gene cluster at Xq27.1 (115). SPANX family encompasses cancer-testis antigens that are epigenetically silenced in normal tissue except testes, while expressed in several human tumors (116, 117). SPANXB1 has been reported to be expressed in melanoma and carcinomas of breast, lung, ovary, colon, and bladder (118–120). In TNBC, SPANXB1 has been shown to promote lung and liver metastasis and be traceable in the circulating extracellular vesicles (120). These data support our findings, and suggest a utility of SPANXB1 as a prognostic biomarker in breast cancer metastasis (120).

Combining the previous studies with insights from our work, we believe that the candidate gene set and individual key genes identified here may be implicated in brain metastasis of breast cancer. The present study may provided new potential biomarkers for BCBM. However, this study has several limitations. Firstly, the screening conducted by us was performed in two TNBC cell lines and the effect of the identified genes on cell migration was only validated in the brain metastatic cell line 231-BR. Although some evidence points to the key genes as potential biomarkers of BCBM, further biological experimental validation and clinical verification along with extensive mechanistic studies are necessary for more accurate and reliable conclusions. Indeed, this is an on-going study in our laboratory with the aim to better clarify and ultimately decipher the underlying mechanism of various key genes. Secondly, although the candidate gene set showed a significantly correlation with brain metastasis-free survival of breast cancer patients from a public dataset, the prognostic value of each individual candidate genes requires further investigation in clinical studies. Since we have not gotten enough brain metastases samples of breast cancer patients from public databases, we have already set up a reliable clinical source in collaboration with some local hospitals and proposed a future study to further investigate the effects of our selected genes from a clinical perspective.

## Conclusion

In the present study, we identified candidate genes that may play roles in BCBM through a series of bioinformatic analyses and wet-lab experiments. The identified genes showed an elevated expression in brain metastatic 231-BR and a prognostic value in patients with BCBM. Among them, KRT19, FKBP10, GSK3B and SPANXB1 were identified as key genes based on their roles in migration of 231-BR. Furthermore, the key genes showed a correlation with the infiltration of major immune cells in the brain TME, suggesting possible roles of them in regulation of immune response in brain TME. Therefore, the present work may provide new potential biomarkers for BCBM.

## Future Directions

Several future directions can be envisioned. The involvement of the identified genes in BCBM demonstrated utility for the identification of biomarkers or potential drug targets for BCBM treatment. Screening brain penetrable compounds targeting these genes may be a promising way for BCBM drug discovery. For example, GSK3B has been studied as a target for drug discovery in the treatment of nervous system disorders (121, 122), and a brain penetrable and orally active GSK3 inhibitor has been reported as a clinical candidate for Alzheimer’s disease and progressed into Phase 1 clinical trials (122). These findings in conjunction with our findings, suggest new indications for such compounds in BCBM.

## Data Availability Statement

The datasets presented in this study can be found in online repositories. The names of the repository/repositories and accession number(s) can be found in the article/**Supplementary Material**.

## Author Contributions

The experiment was designed by all authors. LW and YG performed the bioinformatics analysis and wrote the manuscript. DZ, QW, LL, and TL conducted the experimental part. All authors contributed to the article and approved the submitted version.

## Funding

The present study was supported by National Natural Science Foundation of China (grant no. 81602532); Beijing Natural Science Foundation (grant no.5202004 and 5214022). Support Project of High-level Teachers in Beijing Municipal Universities in the Period of 13th Five-year Plan (grant no. IDHT20170516).

## Conflict of Interest

The authors declare that the research was conducted in the absence of any commercial or financial relationships that could be construed as a potential conflict of interest.

## Publisher’s Note

All claims expressed in this article are solely those of the authors and do not necessarily represent those of their affiliated organizations, or those of the publisher, the editors and the reviewers. Any product that may be evaluated in this article, or claim that may be made by its manufacturer, is not guaranteed or endorsed by the publisher.
