# Supplementary material for: Screening and Identification of Novel Potential Biomarkers for Breast Cancer Brain Metastases
Source: Front Oncol. 2022 Jan 13;11:784096. doi: 10.3389/fonc.2021.784096 (PMC8792448; doi:10.3389/fonc.2021.784096)
Supplement: Supplementary file 1 [file DataSheet_1.zip › Data Sheet 1-corrected/Supplementary Table 9 Relationship between KRT19, FKBP10 and GSK3B expression and clinicopathological features from TCGA breast cancer cohort.docx]

Supplementary Table 9: Relationship betweenKRT19, FKBP10 and GSK3B expression and clinicopathological features from TCGA breast cancer cohort

(n = 1083)

| **Characteristic** |  | **KRT19** |  |  |  | **FKBP10** |  |  |  | **GSK3B** |  |
| --- | --- | --- | --- | --- | --- | --- | --- | --- | --- | --- | --- |
|  | **Low expression** | **High expression** | **P value** |  | **Low expression** | **High expression** | **P value** |  | **Low expression** | **High expression** | **P value** |
| n | 541 | 542 |  |  | 541 | 542 |  |  | 541 | 542 |  |
| T stage, n (%) |  |  | 0.429 |  |  |  | 0.896 |  |  |  | 0.165 |
| T1 | 132 (12.2%) | 145 (13.4%) |  |  | 144 (13.3%) | 133 (12.3%) |  |  | 130 (12%) | 147 (13.6%) |  |
| T2 | 324 (30%) | 305 (28.2%) |  |  | 310 (28.7%) | 319 (29.5%) |  |  | 319 (29.5%) | 310 (28.7%) |  |
| T3 | 64 (5.9%) | 75 (6.9%) |  |  | 69 (6.4%) | 70 (6.5%) |  |  | 77 (7.1%) | 62 (5.7%) |  |
| T4 | 20 (1.9%) | 15 (1.4%) |  |  | 17 (1.6%) | 18 (1.7%) |  |  | 13 (1.2%) | 22 (2%) |  |
| N stage, n (%) |  |  | 0.937 |  |  |  | 0.499 |  |  |  | 0.320 |
| N0 | 262 (24.6%) | 252 (23.7%) |  |  | 268 (25.2%) | 246 (23.1%) |  |  | 270 (25.4%) | 244 (22.9%) |  |
| N1 | 178 (16.7%) | 180 (16.9%) |  |  | 178 (16.7%) | 180 (16.9%) |  |  | 169 (15.9%) | 189 (17.8%) |  |
| N2 | 59 (5.5%) | 57 (5.4%) |  |  | 56 (5.3%) | 60 (5.6%) |  |  | 53 (5%) | 63 (5.9%) |  |
| N3 | 36 (3.4%) | 40 (3.8%) |  |  | 33 (3.1%) | 43 (4%) |  |  | 40 (3.8%) | 36 (3.4%) |  |
| M stage, n (%) |  |  | 0.191 |  |  |  | 0.746 |  |  |  | 0.636 |
| M0 | 472 (51.2%) | 430 (46.6%) |  |  | 462 (50.1%) | 440 (47.7%) |  |  | 432 (46.9%) | 470 (51%) |  |
| M1 | 7 (0.8%) | 13 (1.4%) |  |  | 9 (1%) | 11 (1.2%) |  |  | 8 (0.9%) | 12 (1.3%) |  |
| Pathologic stage, n (%) |  |  | 0.098 |  |  |  | 0.512 |  |  |  | 0.811 |
| Stage I | 83 (7.8%) | 98 (9.2%) |  |  | 98 (9.2%) | 83 (7.8%) |  |  | 91 (8.6%) | 90 (8.5%) |  |
| Stage II | 324 (30.6%) | 295 (27.8%) |  |  | 312 (29.4%) | 307 (29%) |  |  | 311 (29.3%) | 308 (29.1%) |  |
| Stage III | 118 (11.1%) | 124 (11.7%) |  |  | 114 (10.8%) | 128 (12.1%) |  |  | 123 (11.6%) | 119 (11.2%) |  |
| Stage IV | 5 (0.5%) | 13 (1.2%) |  |  | 8 (0.8%) | 10 (0.9%) |  |  | 7 (0.7%) | 11 (1%) |  |
| Race, n (%) |  |  | **0.041** |  |  |  | 0.109 |  |  |  | **0.017** |
| Asian | 30 (3%) | 30 (3%) |  |  | 36 (3.6%) | 24 (2.4%) |  |  | 25 (2.5%) | 35 (3.5%) |  |
| Black or African American | 77 (7.7%) | 104 (10.5%) |  |  | 81 (8.1%) | 100 (10.1%) |  |  | 109 (11%) | 72 (7.2%) |  |
| White | 399 (40.1%) | 354 (35.6%) |  |  | 379 (38.1%) | 374 (37.6%) |  |  | 380 (38.2%) | 373 (37.5%) |  |
| Age, n (%) |  |  | 0.081 |  |  |  | 0.484 |  |  |  | 0.833 |
| <=60 | 315 (29.1%) | 286 (26.4%) |  |  | 294 (27.1%) | 307 (28.3%) |  |  | 298 (27.5%) | 303 (28%) |  |
| >60 | 226 (20.9%) | 256 (23.6%) |  |  | 247 (22.8%) | 235 (21.7%) |  |  | 243 (22.4%) | 239 (22.1%) |  |
| Histological type, n (%) |  |  | **0.014** |  |  |  | 0.264 |  |  |  | **< 0.001** |
| Infiltrating Ductal Carcinoma | 393 (40.2%) | 379 (38.8%) |  |  | 394 (40.3%) | 378 (38.7%) |  |  | 344 (35.2%) | 428 (43.8%) |  |
| Infiltrating Lobular Carcinoma | 84 (8.6%) | 121 (12.4%) |  |  | 95 (9.7%) | 110 (11.3%) |  |  | 147 (15%) | 58 (5.9%) |  |
| PR status, n (%) |  |  | **< 0.001** |  |  |  | **< 0.001** |  |  |  | 0.941 |
| Negative | 214 (20.7%) | 128 (12.4%) |  |  | 204 (19.7%) | 138 (13.3%) |  |  | 174 (16.8%) | 168 (16.2%) |  |
| Indeterminate | 2 (0.2%) | 2 (0.2%) |  |  | 3 (0.3%) | 1 (0.1%) |  |  | 2 (0.2%) | 2 (0.2%) |  |
| Positive | 296 (28.6%) | 392 (37.9%) |  |  | 305 (29.5%) | 383 (37%) |  |  | 345 (33.4%) | 343 (33.2%) |  |
| ER status, n (%) |  |  | **< 0.001** |  |  |  | **< 0.001** |  |  |  | 0.269 |
| Negative | 171 (16.5%) | 69 (6.7%) |  |  | 149 (14.4%) | 91 (8.8%) |  |  | 127 (12.3%) | 113 (10.9%) |  |
| Indeterminate | 1 (0.1%) | 1 (0.1%) |  |  | 2 (0.2%) | 0 (0%) |  |  | 0 (0%) | 2 (0.2%) |  |
| Positive | 341 (32.9%) | 452 (43.7%) |  |  | 362 (35%) | 431 (41.6%) |  |  | 394 (38.1%) | 399 (38.6%) |  |
| HER2 status, n (%) |  |  | 0.932 |  |  |  | 0.122 |  |  |  | 0.274 |
| Negative | 299 (41.1%) | 259 (35.6%) |  |  | 279 (38.4%) | 279 (38.4%) |  |  | 284 (39.1%) | 274 (37.7%) |  |
| Indeterminate | 6 (0.8%) | 6 (0.8%) |  |  | 4 (0.6%) | 8 (1.1%) |  |  | 8 (1.1%) | 4 (0.6%) |  |
| Positive | 86 (11.8%) | 71 (9.8%) |  |  | 90 (12.4%) | 67 (9.2%) |  |  | 72 (9.9%) | 85 (11.7%) |  |
| PAM50, n (%) |  |  | **< 0.001** |  |  |  | **< 0.001** |  |  |  | **< 0.001** |
| Normal | 27 (2.5%) | 13 (1.2%) |  |  | 28 (2.6%) | 12 (1.1%) |  |  | 32 (3%) | 8 (0.7%) |  |
| LumA | 232 (21.4%) | 330 (30.5%) |  |  | 256 (23.6%) | 306 (28.3%) |  |  | 306 (28.3%) | 256 (23.6%) |  |
| LumB | 83 (7.7%) | 121 (11.2%) |  |  | 88 (8.1%) | 116 (10.7%) |  |  | 73 (6.7%) | 131 (12.1%) |  |
| Her2 | 52 (4.8%) | 30 (2.8%) |  |  | 60 (5.5%) | 22 (2%) |  |  | 32 (3%) | 50 (4.6%) |  |
| Basal | 147 (13.6%) | 48 (4.4%) |  |  | 109 (10.1%) | 86 (7.9%) |  |  | 98 (9%) | 97 (9%) |  |
| Menopause status, n (%) |  |  | 0.065 |  |  |  | 0.566 |  |  |  | 0.772 |
| Pre | 126 (13%) | 103 (10.6%) |  |  | 111 (11.4%) | 118 (12.1%) |  |  | 108 (11.1%) | 121 (12.4%) |  |
| Peri | 25 (2.6%) | 15 (1.5%) |  |  | 18 (1.9%) | 22 (2.3%) |  |  | 19 (2%) | 21 (2.2%) |  |
| Post | 340 (35%) | 363 (37.3%) |  |  | 362 (37.2%) | 341 (35.1%) |  |  | 350 (36%) | 353 (36.3%) |  |
| radiation_therapy, n (%) |  |  | 0.678 |  |  |  | 0.862 |  |  |  | 0.898 |
| No | 217 (22%) | 217 (22%) |  |  | 216 (21.9%) | 218 (22.1%) |  |  | 221 (22.4%) | 213 (21.6%) |  |
| Yes | 268 (27.2%) | 285 (28.9%) |  |  | 271 (27.5%) | 282 (28.6%) |  |  | 285 (28.9%) | 268 (27.2%) |  |
| OS event, n (%) |  |  | 0.250 |  |  |  | 0.671 |  |  |  | 0.548 |
| Alive | 458 (42.3%) | 473 (43.7%) |  |  | 468 (43.2%) | 463 (42.8%) |  |  | 469 (43.3%) | 462 (42.7%) |  |
| Dead | 83 (7.7%) | 69 (6.4%) |  |  | 73 (6.7%) | 79 (7.3%) |  |  | 72 (6.6%) | 80 (7.4%) |  |
| DSS event, n (%) |  |  | 0.458 |  |  |  | 0.538 |  |  |  | 0.503 |
| Alive | 482 (45.3%) | 496 (46.7%) |  |  | 489 (46%) | 489 (46%) |  |  | 492 (46.3%) | 486 (45.7%) |  |
| Dead | 46 (4.3%) | 39 (3.7%) |  |  | 39 (3.7%) | 46 (4.3%) |  |  | 39 (3.7%) | 46 (4.3%) |  |
| PFI event, n (%) |  |  | 0.210 |  |  |  | 0.732 |  |  |  | 0.219 |
| Alive | 460 (42.5%) | 476 (44%) |  |  | 470 (43.4%) | 466 (43%) |  |  | 475 (43.9%) | 461 (42.6%) |  |
| Dead | 81 (7.5%) | 66 (6.1%) |  |  | 71 (6.6%) | 76 (7%) |  |  | 66 (6.1%) | 81 (7.5%) |  |
|  |  |  |  |  |  |  |  |  |  |  |  |
