# Supplementary figures and images for: Screening and Identification of Novel Potential Biomarkers for Breast Cancer Brain Metastases
Source: Front Oncol. 2022 Jan 13;11:784096. doi: 10.3389/fonc.2021.784096 (PMC8792448; doi:10.3389/fonc.2021.784096)

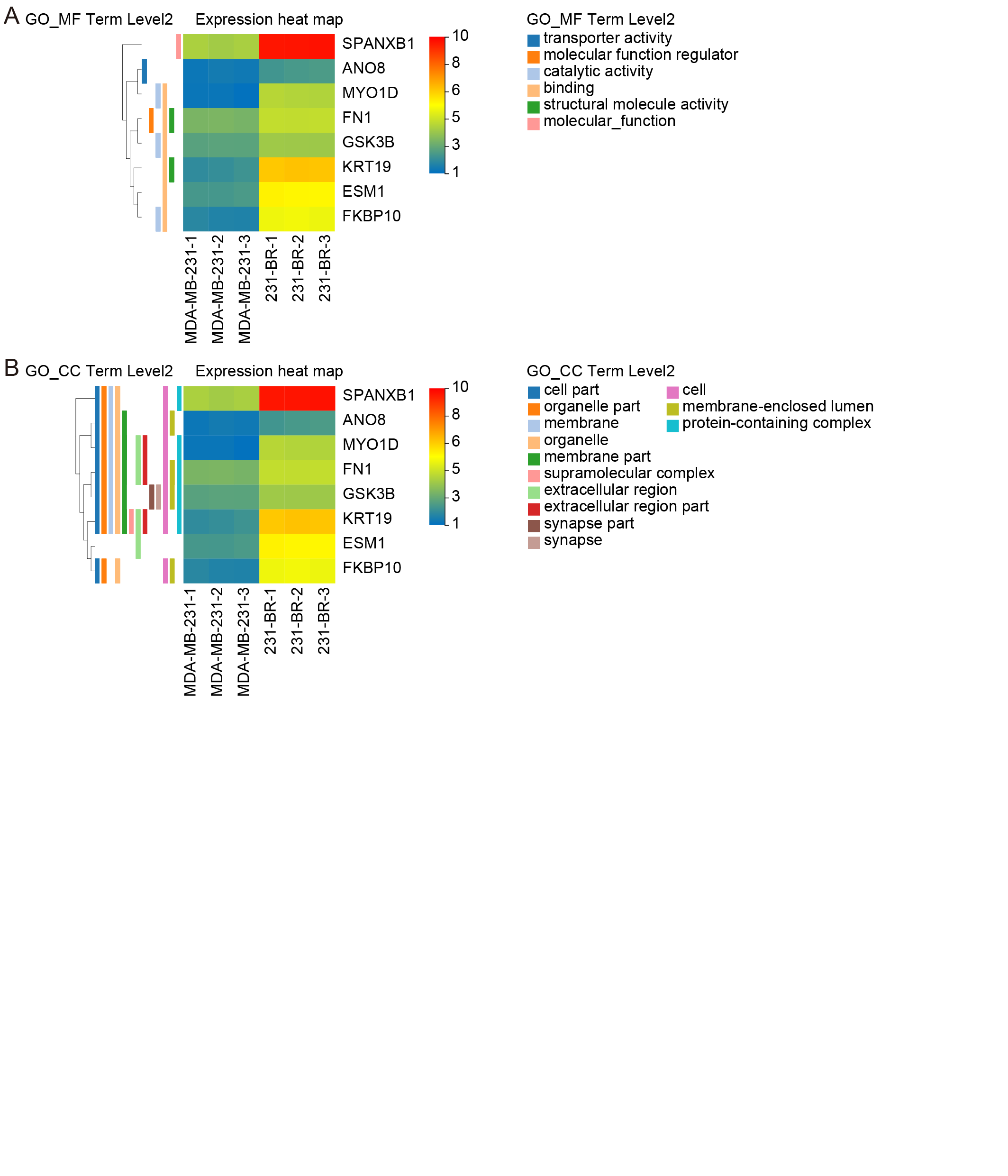

Supplement: Supplementary file 2 [file Image_1.tif]

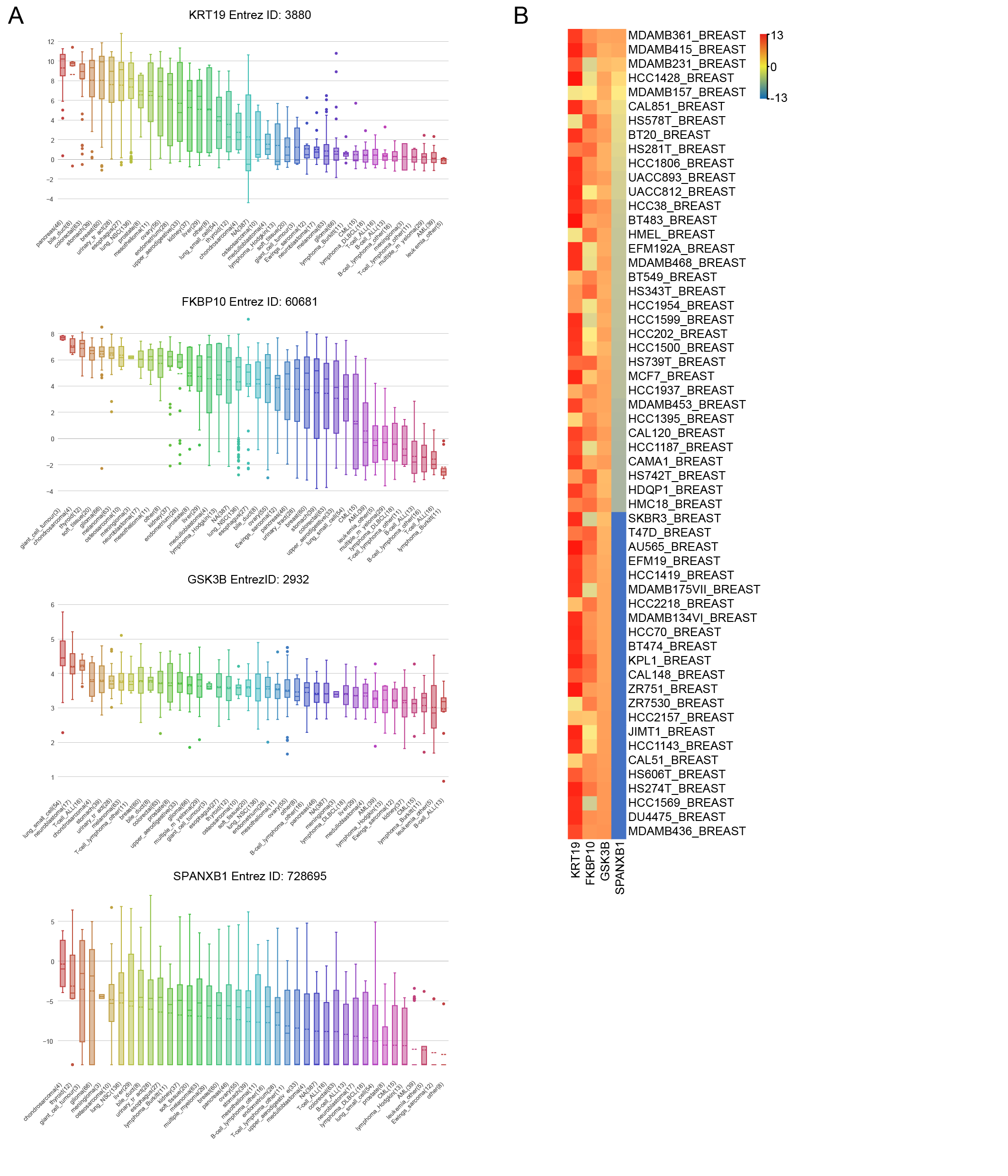

Supplement: Supplementary file 3 [file Image_2.tif]

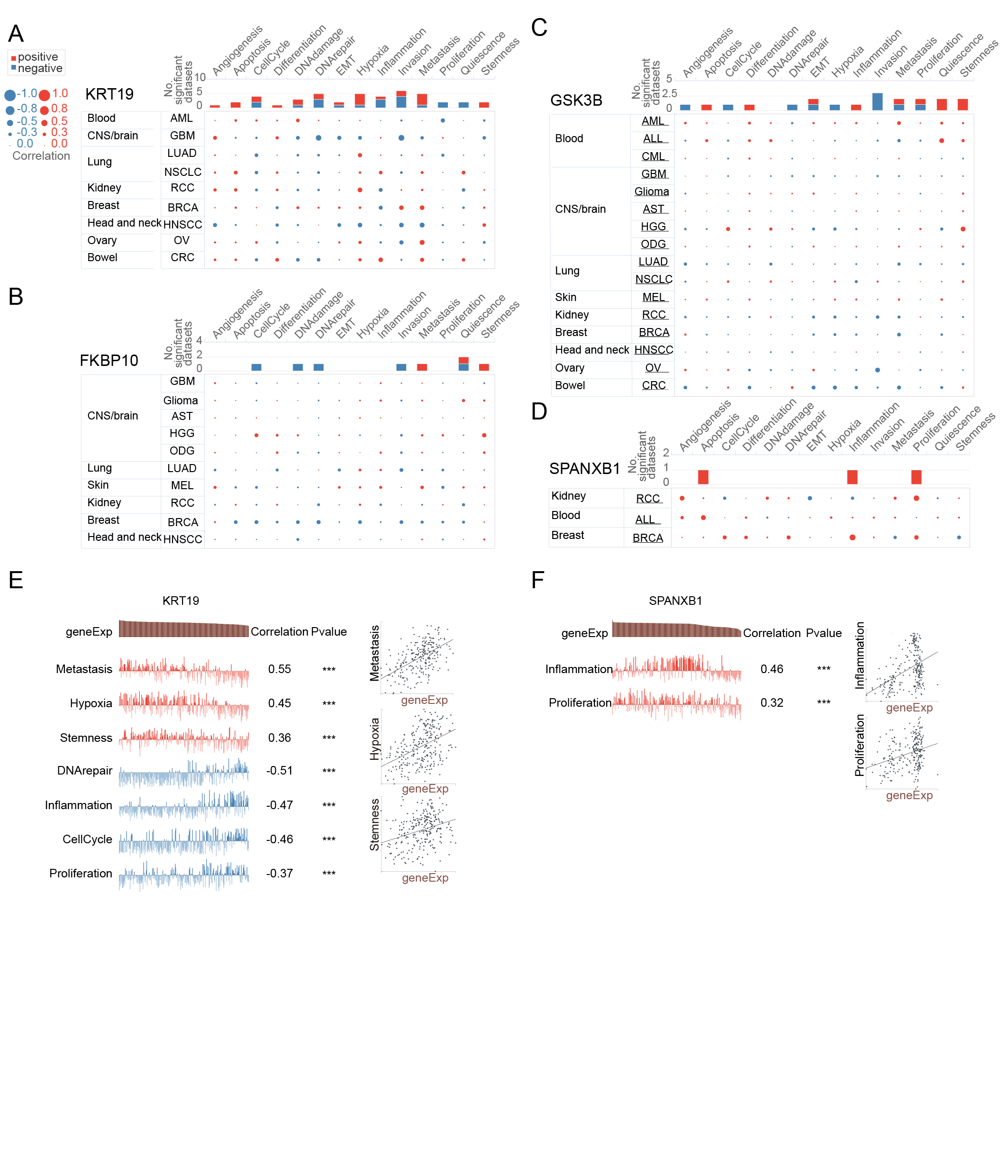

Supplement: Supplementary file 4 [file Image_3.tif]
